# Opioids for breathlessness: psychological and neural factors influencing response variability

**DOI:** 10.1183/13993003.00275-2019

**Published:** 2019-09-19

**Authors:** Sara J. Abdallah, Olivia K. Faull, Vishvarani Wanigasekera, Sarah L. Finnegan, Dennis Jensen, Kyle T.S. Pattinson

**Affiliations:** 1 Clinical Exercise & Respiratory Physiology Laboratory, Dept of Kinesiology & Physical Education, McGill University, Montréal, QC, Canada; 2 Wellcome Centre for Integrative Neuroimaging and Nuffield Division of Anaesthetics, Nuffield Dept of Clinical Neurosciences, University of Oxford, Oxford, UK; 3 Joint first authors

## Abstract

Chronic breathlessness is a multidimensional and aversive symptom, which is often poorly explained by underlying pathophysiology [1]. For many sufferers, breathlessness is refractory to maximal medical therapies that target disease processes [2]. However, opioids are thought to be a possible therapeutic avenue to treat symptomology independently of disease [3]. Importantly, research in chronic pain has demonstrated that qualities such as anxiety and depression (collectively termed negative affect here) can both exacerbate symptoms [4] and reduce opioid efficacy [5, 6]. Therefore, it may be pertinent to consider such behavioural factors when contemplating the use of opioids for breathlessness.

## Abstract 2

**Diminished opioid efficacy in the treatment of breathlessness is related to negative affect and anticipatory brain activity in the anterior cingulate and medial prefrontal cortex.** http://bit.ly/2LXyyDo

To the Editor:

Chronic breathlessness is a multidimensional and aversive symptom, which is often poorly explained by underlying pathophysiology [1]. For many sufferers, breathlessness is refractory to maximal medical therapies that target disease processes [2]. However, opioids are thought to be a possible therapeutic avenue to treat symptomology independently of disease [3]. Importantly, research in chronic pain has demonstrated that qualities such as anxiety and depression (collectively termed negative affect here) can both exacerbate symptoms [4] and reduce opioid efficacy [5, 6]. Therefore, it may be pertinent to consider such behavioural factors when contemplating the use of opioids for breathlessness.

According to the Bayesian brain hypothesis, perception (*e.g.* breathlessness) is the result of a delicate balance between the brain's set of expectations and beliefs (collectively known as priors), and incoming sensory information [7, 8]. An individual's priors are shaped by previous experiences and learned behaviours. For example, if climbing a flight of stairs triggers severe breathlessness, an individual may “expect” to experience severe breathlessness during subsequent stair climbing. Negative affect may act as a moderator within this perceptual system [7–10], altering the balance between priors and sensory inputs to influence symptom perception. Therefore, the relative contribution of sensory inputs and priors (which are thought to be generated in several brain areas, including the anterior cingulate cortex) to overall symptom perception may be important when considering opioid responsiveness for relief of breathlessness.

Abdallah *et al*. [3] demonstrated that 11 out of 20 adults with advanced COPD reported clinically significant relief of exertional breathlessness (defined as a decrease by ≥1 Borg unit) following single-dose administration of immediate-release oral morphine. While the authors were unable to elucidate the physiological mechanisms underlying opioid response variability, they speculated that unmeasured differences in “conditioned anticipatory/associative learning” played a role. The aim of the present study was to test this hypothesis and determine if a relationship exists between physiological factors, behavioural measures of negative affect and opioid responsiveness for relief of breathlessness. Furthermore, we wanted to test if individual opioid responsiveness was related to any differences in neural activity during anticipation of breathlessness. We reanalysed data from Abdallah *et al*. [3] and a behavioural and functional neuroimaging dataset in healthy volunteers by Hayen *et al*. [11], where laboratory-induced breathlessness was manipulated with the opioid remifentanil. As with Abdallah *et al*. [3], Hayen *et al*. [11] observed variability in opioid responsiveness, with 9 of out 19 subjects reporting a remifentanil-induced decrease in breathlessness by ≥10 mm on a 100-mm visual analogue scale. This parallel approach allowed us to verify associations observed in a clinical population in an independent sample that were free of the confounds of chronic disease.

For a complete description of the study design, data acquisition and analyses, please see the original studies [3, 11]. In Abdallah *et al*. [3], 20 participants with COPD completed two sessions, where physiological and perceptual parameters were measured during constant-load cardiopulmonary cycle exercise testing (morphine 0.1 mg·kg^−1^ or saline placebo, randomised order). Intensity and unpleasantness of breathlessness were rated using Borg's modified 0–10 category ratio scale at rest and during exercise [12]. In Hayen *et al*. [11], 19 healthy participants underwent two functional magnetic resonance imaging scans, wherein breathlessness was induced using inspiratory resistive loading combined with mild hypercapnia (remifentanil 0.7 ng·mL^−1^ target controlled infusion or saline placebo, counterbalanced order). Participants also underwent a delay-conditioning paradigm before the scanning visits, wherein they learned associations between three visual cues presented on a screen and three conditions: mild inspiratory load (approximately −3 cmH_2_O), strong load (approximately −12 cmH_2_O) and unloaded breathing. A cued anticipation period of 8 s preceded each loading condition. Participants rated the intensity and unpleasantness of their breathlessness using a visual analogue scale (0–100 mm). The change in all scores was calculated as opioid minus placebo.

In both datasets, a hierarchical cluster analysis (MATLAB 2013a; MathWorks Inc., Natick, MA, USA) was performed on questionnaires, breathlessness ratings and physiological measures; all included measures are listed in figure 1. In the COPD dataset, the hierarchical cluster analysis supported the existence of three distinct clusters of variables, verified by the elbow method; a validated cluster threshold technique that determines the number of clusters in a dataset (see figure 1). Cluster A included items that predominantly represented responses to opioid administration, breathlessness and affective measures, and was therefore designated as a “response and state-trait affect” cluster. Both Clusters B and C contained affective and subjective measures at rest and during the placebo condition and were designated as “baseline” clusters. See figure 1 for the complete list of variables induced in each sub-cluster.

**FIGURE 1:**
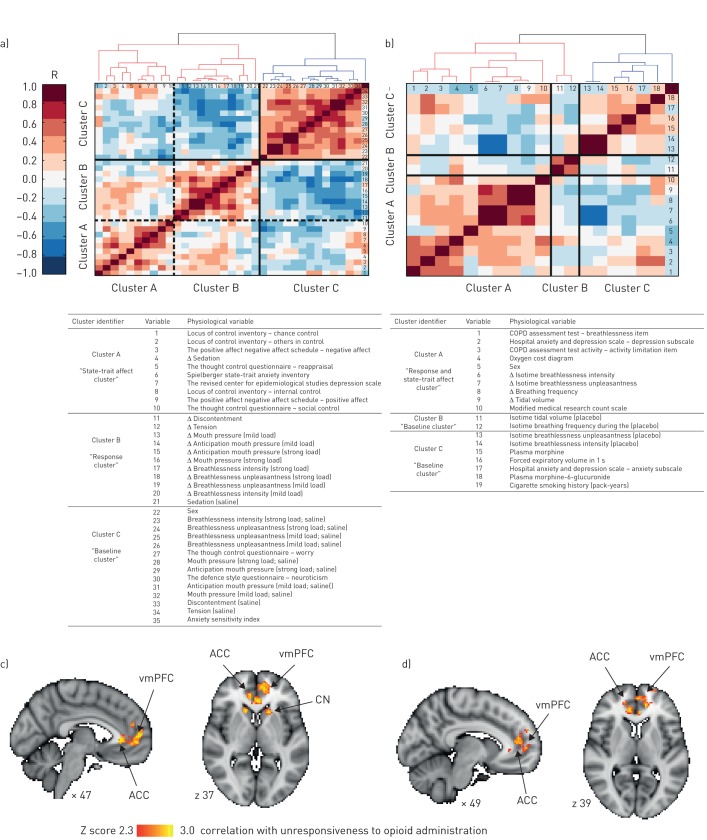
The hierarchical cluster analysis allowed us to explore the possible relationships between the magnitude of opioid-induced relief of breathlessness, behavioural measures and physiological traits. Variables were first aligned such that larger values represented more negative properties (*via* multiplication of relevant variables by −1). All measures were then individually normalised *via* Z-transformation, to allow accurate variable comparisons and distance calculations. The distance between neighbouring branches indicates the relative similarity of two measures. Mathematically distinct clusters were determined *via* the “elbow method”, with a minimum intra-cluster correlation coefficient of 0.3 between the variables, and further cluster divisions were considered utilising *a priori* knowledge and visual inspection of the dendrogram structure. The elbow method is a validated clustering technique in which the percentage of explained variance is described as a function of the number of clusters. Considering the variable set as initially one large cluster, the algorithm then divides the variables into increasing numbers of clusters. With each additional cluster, the percentage of explained variance is expected to increase. While initially this increase is sharp, after a certain number of clusters the gain will become marginal. When this relationship is plotted, as the sum of intra-cluster distance against cluster number, the point at which additional clusters add only marginally to the explained variance can be seen as a sharp bend or elbow in the graph. The number of clusters corresponding to this elbow point is thus the number of most statistically distinct clusters in the dendrogram. Clustergram of physiological and behavioural variables in a) healthy volunteers and b) participants with COPD. Identified hard cluster boundaries (*via* the elbow method) are denoted in solid lines, whilst sub-clusters (*via* visual inspection) are denoted with dashed lines. Tables identify the physiological and behavioural variables included in each of the sub-clusters. The change (Δ) in all scores was calculated as: opioid minus placebo. In the COPD dataset, physiological and perceptual responses were evaluated during exercise at isotime, defined as the highest equivalent 2-min interval of exercise completed by each participant after oral morphine and placebo. c, d) We explored how brain activity associated with anticipation of breathlessness (during the saline placebo condition) may relate to an individual's “opioid efficacy” for the treatment of breathlessness. This analysis allowed us to determine if there was an association between the activity of prior rich brain regions and opioid responsiveness. The group of items that formed Cluster B within the hierarchical cluster analysis on the healthy volunteers were used to define overall opioid efficacy (*i.e.* items that represented opioid-induced changes in physiological and subjective measures). We employed a principal component analysis (MATLAB 2013a; MathWorks Inc., Natick, MA, USA) on this group of variables, and the resulting individual scores were included within a group functional magnetic resonance imaging analysis of the saline placebo condition only, using a general linear model (Z>2.3, whole brain corrected p<0.05). The resulting mean bold changes identified during anticipation of the c) mild and d) strong breathlessness challenge. The image consists of a colour-rendered statistical map superimposed on a standard (MNI 2×2×2) brain. Significant regions are displayed with a threshold Z>2.3, using a cluster probability threshold of p<0.05. ACC: anterior cingulate cortex; CN: caudate nucleus; vmPFC: ventromedial prefrontal cortex.

In the healthy volunteer dataset, the elbow method initially supported the existence of two distinct clusters (figure 1). Upon visual inspection, the larger cluster could clearly be split into two distinct and related clusters (figure 1). We designated Cluster A as a “state-trait affect” cluster, Cluster B a “response” cluster and Cluster C a “baseline” cluster (figure 1). In both datasets, the predominant state-trait affect and response clusters were more closely related to each other than to the baseline cluster. Importantly, the association between the state-trait affect and response clusters indicated that worse affective scores corresponded to a smaller degree of opioid-induced relief of breathlessness.

These behavioural findings suggest that opioid responsiveness is inversely associated with the collective coexisting weight of affective moderators. This work aligns with previous findings in chronic pain, where it has been found that in addition to less effective analgesia, negative affective qualities are associated with dose escalation [13] and greater difficulty in reducing opioid medication use [14]. Interestingly, the cluster structure revealed in the COPD participants was conceptually consistent with that found in the healthy volunteers. Free of the confounds of respiratory disease, the results in these healthy individuals suggest that even subtle variations in affective traits may have measurable effects upon opioid responsiveness.

To extend these behavioural findings and further explore the potential influence of prior expectations, we then investigated how brain activity during anticipation of breathlessness (saline placebo condition) may relate to an individual's “opioid efficacy” using the brain imaging data from Hayen *et al*. [11]. This analysis revealed significant anticipatory brain activity that correlated with opioid unresponsiveness in the anterior cingulate cortex and ventromedial prefrontal cortex prior to both mild and strong loading; and in the caudate nucleus prior to mild loading only (figure 1). That is, the greater the activity in these brain regions during anticipation of breathlessness under placebo conditions, the smaller the degree of opioid-induced relief of breathlessness.

Interestingly, the anterior cingulate cortex and ventromedial prefrontal cortex are thought to be part of a brain network involved in generating predictions on emotional state and bodily awareness [8, 15]. When anticipating breathlessness, individuals with greater brain activity in these regions were less likely to experience meaningful opioid-induced relief of breathlessness, and therefore are potentially more resistant to opioid therapy. If this brain activity is related to negative affective properties, these might influence breathlessness perception by more heavily weighting the brain's perceptual system towards learned priors during anticipation of breathlessness [7]. For example, in anticipation of climbing a set of stairs, an individual with high negative affect may have worse breathlessness expectations relative to an individual with less negative affect. In turn, and despite receiving the same sensory afferent inputs when climbing the stairs, the individual with more negative affect may be less responsive to opioid therapy as their breathlessness perception is more rigidly attracted towards their breathlessness expectations (*i.e.* strong, precise priors).

Finally, whilst this neuroimaging work was completed in healthy volunteers, previous neuroimaging studies have evaluated the relationship between learned associations and relief of breathlessness in COPD. In contrast to our findings with opioids, Herigstad *et al*. [16] reported that baseline activity in the brain network responsible for generating predictions (*e.g.* anterior cingulate cortex) correlated positively with changes in breathlessness following pulmonary rehabilitation in COPD. Pulmonary rehabilitation is thought to exert its benefits, in part, by re-shaping associations and modulating negative affect [16]. The results of these studies suggest that individuals with strong learned associations (priors) and negative affective comorbidities may be more likely to benefit from treatments such as pulmonary rehabilitation than opioids for relief of breathlessness. It is also possible that individuals with these strong learned associations and negative affective comorbidities may require higher opioid doses to experience adequate relief of breathlessness, as previously demonstrated in pain [5, 13, 14].

This initial, explorative study is limited by its retrospective, cross-sectional nature and small sample sizes, and future work is required to specifically explore and accurately quantify the relationship between negative affective qualities and opioid responsiveness in health and disease. Nevertheless, the datasets by Abdallah *et al*. [3] and Hayen *et al*. [11] allowed us to investigate potential predictors of opioid responsiveness, and to generate hypotheses based on possible neurobiological mechanisms of action. Although additional research is necessary, our results are unique and support the hypothesis that opioids may be less effective for relief of breathlessness among individuals with higher levels of negative affective comorbidities and strong learned associations (priors).

## Shareable PDF

10.1183/13993003.00275-2019.Shareable1 This one-page PDF can be shared freely online.
Shareable PDF ERJ-00275-2019.Shareable
