# Supplementary material for: Opioids for breathlessness: psychological and neural factors influencing response variability
Source: Eur Respir J. 2019 Sep 19;54(3):1900275. doi: 10.1183/13993003.00275-2019 (PMC6751386; doi:10.1183/13993003.00275-2019)
Supplement: Supplementary file 1 [file ERJ-00275-2019.Shareable.pdf]

SHAREABLE PDF

Opioids for breathlessness: psychological and neural factors influencing response variability

Sara J. Abdallah[1,3], Olivia K. Faull[2,3], Vishvarani Wanigasekera[2], Sarah L. Finnegan[2], Dennis Jensen[1] and Kyle T.S. Pattinson[2]

Affiliations: [1]Clinical Exercise & Respiratory Physiology Laboratory, Dept of Kinesiology & Physical Education, McGill University, Montréal, QC, Canada. [2]Wellcome Centre for Integrative Neuroimaging and Nuffield Division of Anaesthetics, Nuffield Dept of Clinical Neurosciences, University of Oxford, Oxford, UK. [3]Joint first authors.

Correspondence: Kyle Pattinson, Nuffield Dept of Clinical Neurosciences, West Wing Level 6, John Radcliffe Hospital, Oxford, OX3 9DU, UK. E-mail: kyle.pattinson@nda.ox.ac.uk

@ERSpublications
Diminished opioid efficacy in the treatment of breathlessness is related to negative affect and anticipatory brain activity in the anterior cingulate and medial prefrontal cortex. http://bit.ly/2LXyyDo

Cite this article as: Abdallah SJ, Faull OK, Wanigasekera V, *et al*. Opioids for breathlessness: psychological and neural factors influencing response variability. *Eur Respir J* 2019; 54: 1900275 [https://doi.org/10.1183/13993003.00275-2019].

This single-page version can be shared freely online.

To the Editor:

Chronic breathlessness is a multidimensional and aversive symptom, which is often poorly explained by underlying pathophysiology [1]. For many sufferers, breathlessness is refractory to maximal medical therapies that target disease processes [2]. However, opioids are thought to be a possible therapeutic avenue to treat symptomology independently of disease [3]. Importantly, research in chronic pain has demonstrated that qualities such as anxiety and depression (collectively termed negative affect here) can both exacerbate symptoms [4] and reduce opioid efficacy [5, 6]. Therefore, it may be pertinent to consider such behavioural factors when contemplating the use of opioids for breathlessness.

Copyright ©ERS 2019 This article is open access and distributed under the terms of the Creative Commons Attribution Licence 4.0.
